# Vitamin D inhibits the proliferation of Oral Squamous Cell Carcinoma by suppressing lncRNA LUCAT1 through the MAPK pathway

**DOI:** 10.7150/jca.45389

**Published:** 2020-08-15

**Authors:** Tingting Jin, Ying Guo, Zixian Huang, Qianyu Zhang, Zhuoshan Huang, Yin Zhang, Zhiquan Huang

**Affiliations:** 1 Department of Oral and Maxillofacial Surgery, Sun Yat-sen Memorial Hospital, Sun Yat-sen University, 107 Yan-jiang Road, Guangzhou, Guangdong Province, China, 510120.; 2 Department of Endocrinology, Sun Yat-sen Memorial Hospital, Sun Yat-sen University, 107 Yan-jiang Road, Guangzhou, Guangdong Province, China, 510120.; 3 Department of Stomatology, Sun Yat-sen Memorial Hospital,Sun Yat-sen University, 107 Yan-jiang Road, Guangzhou, Guangdong Province, China, 510120.; 4 Key Laboratory of Malignant Tumor Epigenetics and Gene Regulation, Research Center of Medicine, Sun Yat-sen Memorial Hospital, Sun Yat-sen University, Guangzhou 510120, P. R. China.

**Keywords:** Vitamin D, Oral squamous cell carcinoma, LUCAT1, Cell proliferation

## Abstract

**Background:** Oral squamous cell carcinoma (OSCC) is the most common type of oral cancer worldwide. Recent studies have suggested that vitamin D (VitD) is associated with a reduced risk of many chronic illnesses, including cancer. However, the role of vitamin D in OSCC has rarely been reported.

**Materials and Methods:** The effect of vitamin D and control treatment were examined by cell clone formation assay. Using RNA-seq, we globally identified VitD-regulated long noncoding RNAs (lncRNAs). The expression of LUCAT1 in OSCC tissues and cell lines was examined by qRT-PCR. The correlation between LUCAT1 expression level and clinicopathological characteristics was analyzed. The biological roles of LUCAT1 in OSCC cell proliferation was determined by CCK8 and cell colony formation. The role of LUCAT1 in OSCC growth was further confirmed by mouse xenograft tumor model. Combined with the literature, the mechanism of action of LUICAT1 was verified by western blot.

**Results:** In this study, we observed that VitD inhibited tumour cell growth in OSCC. We found that lncRNA LUCAT1 was downregulated by VitD and served as an important mediator of VitD in inhibiting OSCC cell proliferation. Moreover, we observed that the expression of LUCAT1 was significantly upregulated in OSCC tissues compared to non-tumour tissues. We further demonstrated that LUCAT1 promoted the proliferation of oral cancer cells by enhancing the activation of the mitogen protein kinase (MAPK) signalling pathway.

**Conclusion:** In summary, our results show that VitD inhibited the growth of OSCC cells through the LUCAT1-MAPK signalling pathway. Our study suggested that VitD could suppress the progression of oral cancer, and LUCAT1 may be a potential tumour marker for the diagnosis and prognosis of OSCC.

## Introduction

According to the latest statistics by the American Cancer Society, oral squamous cell carcinoma (OSCC) and oropharynx carcinoma are the ninth most prevalent cancers worldwide 1. Despite advances in surgical techniques and postoperative radiotherapy, the overall 5-year survival of OSCC patients has not significantly improved in the last 50 years. The 5-year survival rate for OSCC patients is still only approximately 40%-50% [2.3]. The lack of suitable early detection targets and new molecular-targeted antitumour treatments remains a major challenge for OSCC therapy 4. Moreover, the molecular mechanism underlying OSCC development is still unclear. Few targetable genomic lesions have been identified and no molecular therapy is available 5. Therefore, we sought to explore the carcinogenic mechanism of OSCC to find new targets for OSCC treatment.

Vitamin D (VitD) is a derivative of fat-soluble steroids (also known as 1,25-dihydroxy-vitamin D3 (1,25(OH)2D3)) that can be divided into vitamin D2 and vitamin D3. The body acquires VitD mainly through ultraviolet radiation and only a small portion is obtained from food. One of the first described classic effects of VitD is its ability to regulate calcium and phosphorus metabolism and promote bone mineralisation. Increasing epidemiological data suggest an important role of VitD signalling in cancer development and progression, and experimental studies demonstrate that the active VitD metabolite 1α,25-dihydroxy-VitD_3_ (1,25D_3_), has broad antitumour activity 6. 1,25-Dihydroxy-VitD_3_ exerts a suppressive effect on kidney cancer cells via upregulation of FOXO3 7. VitD analogues suppress IGF-I signalling and promote apoptosis in breast cancer cells 8. Our previous research found that VitD can inhibit the progression of adenoid cystic carcinoma and oesophageal squamous cell carcinoma 9, 10. Recently, we further found that VitD promoted the cisplatin sensitivity of oral squamous cell carcinoma 11. All these results indicate that VitD may play an important role in the prevention and treatment of tumours.

Long noncoding RNAs (lncRNAs) are defined as transcripts with a length of at least 200 nt that are unlikely to be translated into proteins. It is reported that only 2% of human genome encodes for proteins 12, the number of lncRNAs is more than twice as many as protein-coding genes 13. LncRNAs have been reported to play crucial roles in multiple biological processes, including tumorigenesis and progression. In recent years, the important role of lncRNAs in tongue cancer has been studied. Dai et al. found high expression of HOTAIR in TSCC. They noted that HOTAIR participates in the regulation of proliferation and apoptosis of TSCC cells 14. Liang et al. found that lncRNA MALAT1 expression was upregulated in tongue tissues and correlated with cervical lymph node metastasis 15. However, there is no research on whether VitD has antitumour effects through lncRNAs in TSCC, and the underlying mechanisms of Vitamin D's anti-tumor effect at the lncRNA layer is still largely unknown.

Here, we report a genome-wide study on vitD-regulated lncRNAs through an integrated analysis of strand-specific RNA-seq and TCGA clinical data. Moreover, we observed that LUCAT1 is an important vitD-regulated lncRNA and that transiently or stably silenced LUCAT1 significantly inhibited OSCC proliferation. These findings reveal that VitD can inhibit the proliferation of OSCC by regulating the LUCAT1/MAPK signalling pathway. Our study provides insights for the links between lncRNAs and Vitamin D effects.

## Materials and Methods

### Antibodies

Mouse monoclonal anti-FLAG (#F3165) was purchased from Sigma. Anti-phospho-p44/42 MAPK (Erk1/2) (Thr202/Tyr204) antibody #9101, anti-p44/42 MAPK (Erk1/2) (137F5) antibody (#4695), anti-rabbit IgG, HRP-linked antibody 7074, and anti-GAPDH antibody (#D16H11) were purchased from Cell Signaling Technology. Antigen Ki-67 (YM6189) was purchased from Immunoway.

### Reagents

The VitD metabolite 1,25D3 (Sigma, USA) was dissolved at a concentration of 400 μM in anhydrous alcohol (AA) for preservation. Immediately prior to use, the stock was diluted to a final concentration of 30 nm in culture medium. Si-RNA LUCAT1 was purchased from GenePharma (Suzhou, China). Other chemicals were purchased from Sigma. All of the culture media (DMEM for CAL-27, DMEM-F12 for SCC-9) and foetal bovine serum (FBS) were purchased from Bioind.

### OSCC sample collection and patient follow-up

To address the research aim, patients seen at the Department of Oral and Maxillofacial Surgery, Sun Yat-sen Memorial Hospital between 2017 and 2018 for the treatment of OSCC were recruited. Inclusion criteria included a pathological diagnosis of OSCC and willingness to participate in the subsequent follow-up. Patients were excluded from the study if they were diagnosed with multiple cancers or other severe diseases. Data on the characteristics of the OSCC patients, including age, gender, tumour differentiation, lymphatic metastasis and clinical stage, were collected. All patients had an appointment at least every season. In addition, patient tumour samples and adjacent noncancerous (ANC) samples were collected. The ANC tissue refers to an area at least 2 cm from the tumour lesion that represents the resection border and is pathologically confirmed as noncancerous tissue.

### Cell culture and transfection

The human oral squamous cell carcinoma cell lines CAL27 and SCC9 and the lentivirus vector packaging cell line HEK293T were obtained from the American Type Culture Collection (ATCC). All OSCC cell lines were routinely cultured in DMEM and DMEM-F12 medium supplemented with 10% FBS in a 37°C humidified incubator containing 5% CO2. All the cell lines were validated by short tandem repeat profiling analysis and were free of mycoplasma contamination.

Transient transfection of OSCC cells was performed using Lipofectamine 3000 (Invitrogen) reagent according to the manufacturer's instructions. For transient transfections using small interfering RNAs (siRNAs), siRNAs targeting LUCAT1 were synthesised by GenePharma (Suzhou, China). The transfection was performed with Lipofectamine 3000 reagent (Invitrogen, Carlsbad, USA) according to the manufacturer's protocol. The siRNA sequences are shown in Table 1.

For stable expression, short hairpin RNA targeting LUCAT1 (shLUCAT1#1 and shLUCAT1#1) and the corresponding control scramble were designed and synthesised by Genepharma (Shanghai, China). Subsequently, RT-qPCR was performed to confirm the correct expression of the stable cell lines. We selected shLUCAT1#2 due to its stronger knockdown effect for the subsequent experiment.

### Immunohistochemistry (IHC)

Immunohistochemical staining was performed according to standard protocols. After deparaffinisation, antigen retrieval was conducted using 10 mM sodium citrate buffer (pH 8.0) in a pressure cooker at full power for 5 min. Briefly, the tissue sections were blocked sequentially with 3% H_2_O_2_ and normal serum and then incubated with primary antibodies at 4°C overnight. The tissue sections were incubated with a biotinylated secondary antibody and conjugated with a streptavidin-HRP complex (ready-to-use SP kit; Zhongshan Co. Beijing, China). Finally, the sections were visualised with 3-3′diaminobenzidine, counterstained with haematoxylin and mounted. The samples were rinsed with phosphate-buffered saline (PBS) between each step.

### Western blot

For protein extraction, the cells were washed twice with cool PBS, harvested by scraping and then lysed in lysis buffer (Beyotime, China). Following centrifugation, the supernatant was collected, and the protein concentration was determined using the BCA Protein Assay Kit (Pierce, USA). For western blotting, cell lysates were electrophoretically separated on an SDS-PAGE gel using a standard protocol. The proteins were then transferred to polyvinylidene fluoride (PVDF) membranes (IPVH00010; Millipore, USA). The membranes were blocked with 5% non-fat milk in Tris-buffered saline containing 0.1% Tween-20 (TBST) for 1 h at room temperature. The blots were then incubated with the antibodies mentioned above at 4°C overnight, washed in TBST and then probed with secondary antibody. Western blot analysis was performed using the band intensity measurements of the blots.

### RNA extraction, real-time quantitative RT-PCR and RNA sequencing

Total RNA was extracted using TRIzol reagent (Takara, Japan) according to the manufacturer's instructions and then reverse transcribed into cDNA using PrimeScript™ RT Master Mix (Takara, Japan) on an ABI 9700 Real-Time PCR system (ABI, USA). The newly synthesised cDNA was then used as a template for the detection of the desired gene. Specifically, 1 μl of cDNA was mixed with TB Green® Premix Ex Taq™ II (Takara, Japan) in a 20-μl reaction. All of the reactions were run in triplicate using the primers described above. The reaction conditions were as follows: 94°C for 2 min, 94°C for 20 s, 58°C for 20 s and 72°C for 20 s, for 40 cycles. The relative expression of mRNA was detected using the Roche LightCycler 480 II Real-time PCR machine (Roche, USA). The primer sequences are shown below in Table 2.

### Cell proliferation assay

CAL27 and SCC9 cells were seeded at a density of 1000 cells per well into 96-well plates in triplicate and cultured for 24 to 72 hours after transfection. Before observation, each well was treated with 10 μL/well of cell counting kit-8 solution (Dojindo, Japan) during the last 4 hours of culture. Then, cell viability was determined by measuring the absorbance of the converted dye at 450 nm. For the colony formation assay, specific numbers of transfected cells were placed into each well of a six-well plate and cultured in medium containing 10% FBS for 15 days, replacing the medium every 5 days. Colonies were fixed with methanol and stained with 0.1% crystal violet (Sigma-Aldrich, St. Louis, MO, USA) in PBS for 15 min. Colony formation was determined by counting the number of stained colonies. For each treatment group, wells were counted in triplicate.

### Statistical analyses

All statistical analyses were conducted using SPSS 22.0 statistical software (IBM, USA). The survival curves were plotted using the Kaplan-Meier method and compared with the log-rank test. Student's *t* test was used to compare the RT-qPCR results, tumour xenograft results, and cell proliferation between the different groups. Unless otherwise noted, quantitative data are expressed as the mean and standard error of the mean (S.E.M). Statistical significance was determined with a paired Student's *t* test. *P<0.05; **P<0.01; ***P<0.001, compared with the control.

## Results

### VitD inhibited cell proliferation in OSCC

**Figure 1:**
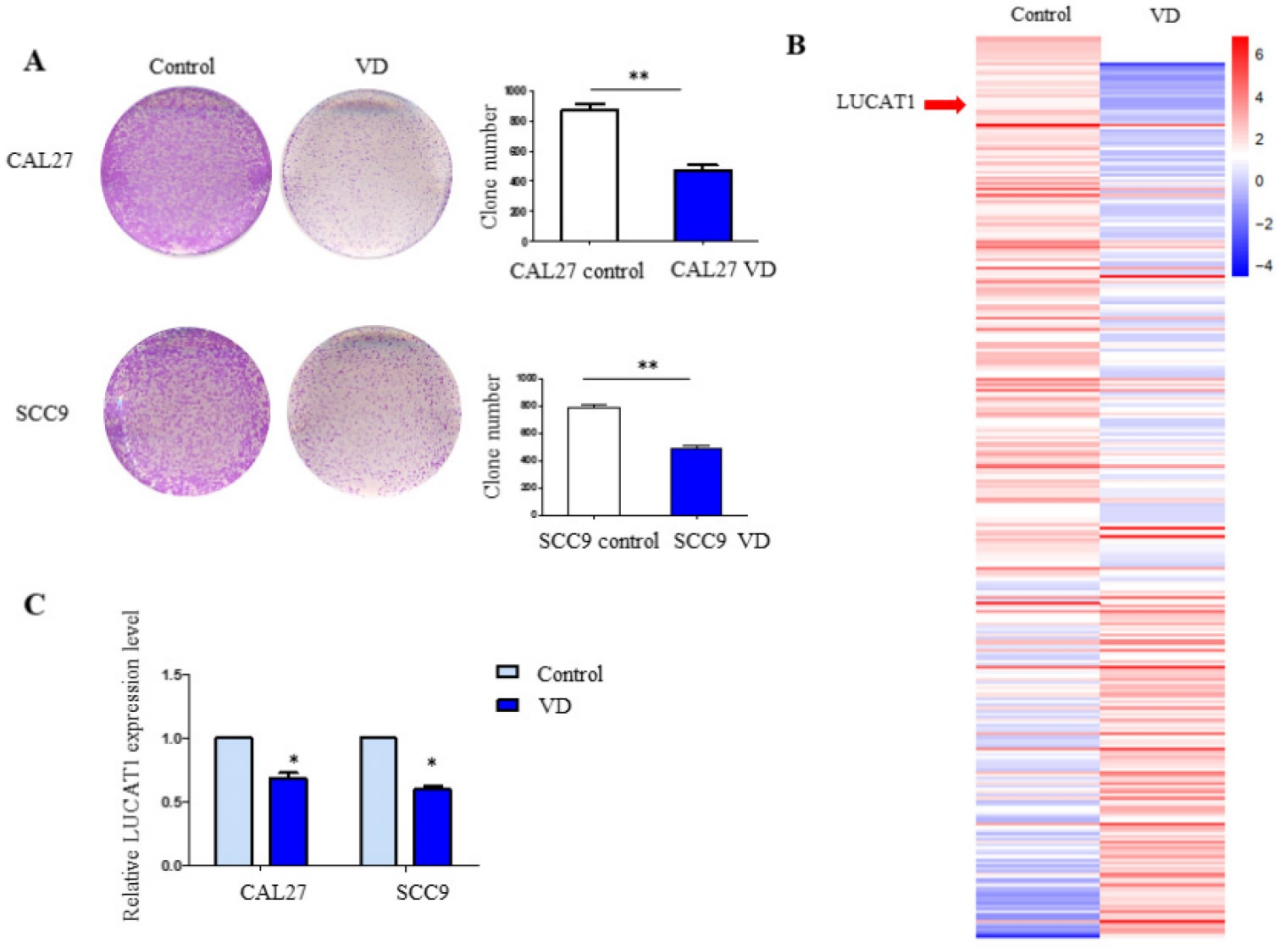
** Vitamin D inhibits TSCC cell proliferation and identification of VitD-regulated lncRNAs. A.** The colony formation assay results in CAL27 and SCC9 cells treated with vitamin D. **B.** Heatmap of high-throughput sequencing data in CAL27 cells treated with PBS (control) or vitamin D (Vit D). **C.** RT-qPCR detection of the expression levels of LUCAT1 with vitamin D treatment in SCC9 and CAL27 cells. Error bars indicate the means ± S.E.M. *P < 0.05, **P < 0.01. ***P < 0.001.

Our previous study demonstrated the role of vitamin D in chemotherapy resistance in OSCC, and we further explored other effects of vitamin D on OSCC. To investigate the effect of LUCAT1 on OSCC cell growth, cell proliferation was measured by colony formation assays. CAL27 and SCC9 cells were pretreated with 30 nM VitD for 3 days, and then a colony formation assay was performed. The results revealed that the colony formation abilities of CAL27 and SCC9 cells pretreated with VitD were decreased (Figure 1A).

### LUCAT1 was the key target in VitD-regulated proliferation of OSCC

We pretreated CAL27 cells with 30 nM VitD for 72 h. Then, total RNA was extracted for high-throughput sequencing. In addition to the changes in protein-coding genes, we found that many lncRNAs showed significant changes after VitD treatment. We further carried out systematic identification of VitD-regulated lncRNAs. We found that 1045 VitD-regulated lncRNAs showed significant differential expression between VitD group and control group in OSCC cell (Table S1). LUCAT1 was one of the most significantly changed VitD-regulated lncRNAs (Figure 1B). We verified the results by RT-qPCR, and similar results were obtained for both CAL27 and SCC9 cells (Figure 1C).

### LUCAT1 was highly expressed in OSCC and associated with clinicopathological parameters

**Figure 2:**
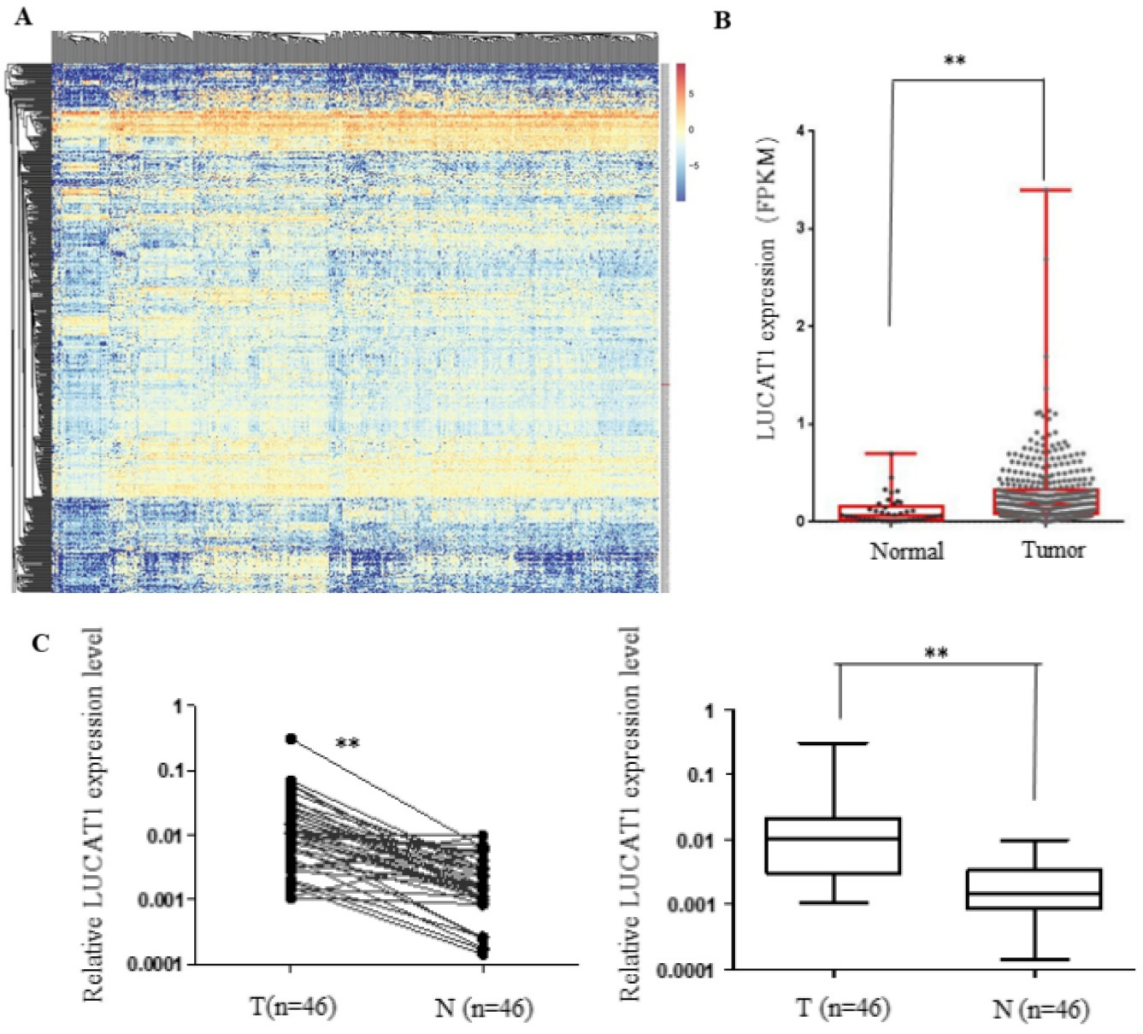
** LUCAT1 expression is increased in TSCC and is associated with clinicpathological Parameters. A, B.** Analysis of RNA-seq data of LUCAT1 in TCGA. **C.** LUCAT1 expression in TSCC tissues (n = 46) increased significantly compared to that in the corresponding non-tumour tissues (n = 46). LUCAT1 expression was detected by RT-PCR and normalised to GAPDH expression. Error bars indicate the means ± S.E.M. *P < 0.05, **P < 0.01. ***P < 0.001.

The expression levels of abnormally expressed lncRNAs in human head and neck cancer samples was evaluated using TCGA RNA-seq data. The differentially expressed lncRNAs were screened, and the most significantly dysregulated lncRNAs are shown in the heatmap (Figure 2A). In addition, the expression of LUCAT1 was significantly upregulated in head and neck cancer tissues compared to normal tissues in TCGA datasets (Figure 2B). We further performed RT-qPCR to measure the expression pattern of LUCAT1 in our own cohort of 46 pairs of OSCC tissues and adjacent noncancerous oral tissues, and the results suggested that LUCAT1 was highly expressed in OSCC tissues compared to normal tissues (*p* < 0.01). To explore the clinical significance of LUCAT1 in OSCC, the 46 patients with OSCC were divided into several groups according to their postoperative pathology. As shown in Table 3, there was a statistically significant difference in the expression of LUCAT1 between different tumour sizes. Tumour size was associated with the expression of LUCAT1. However, there was no significant difference between LUCAT1 expression and other clinicopathological factors.

### Downregulation of LUCAT1 expression inhibited OSCC cell growth *in vitro*

**Figure 3:**
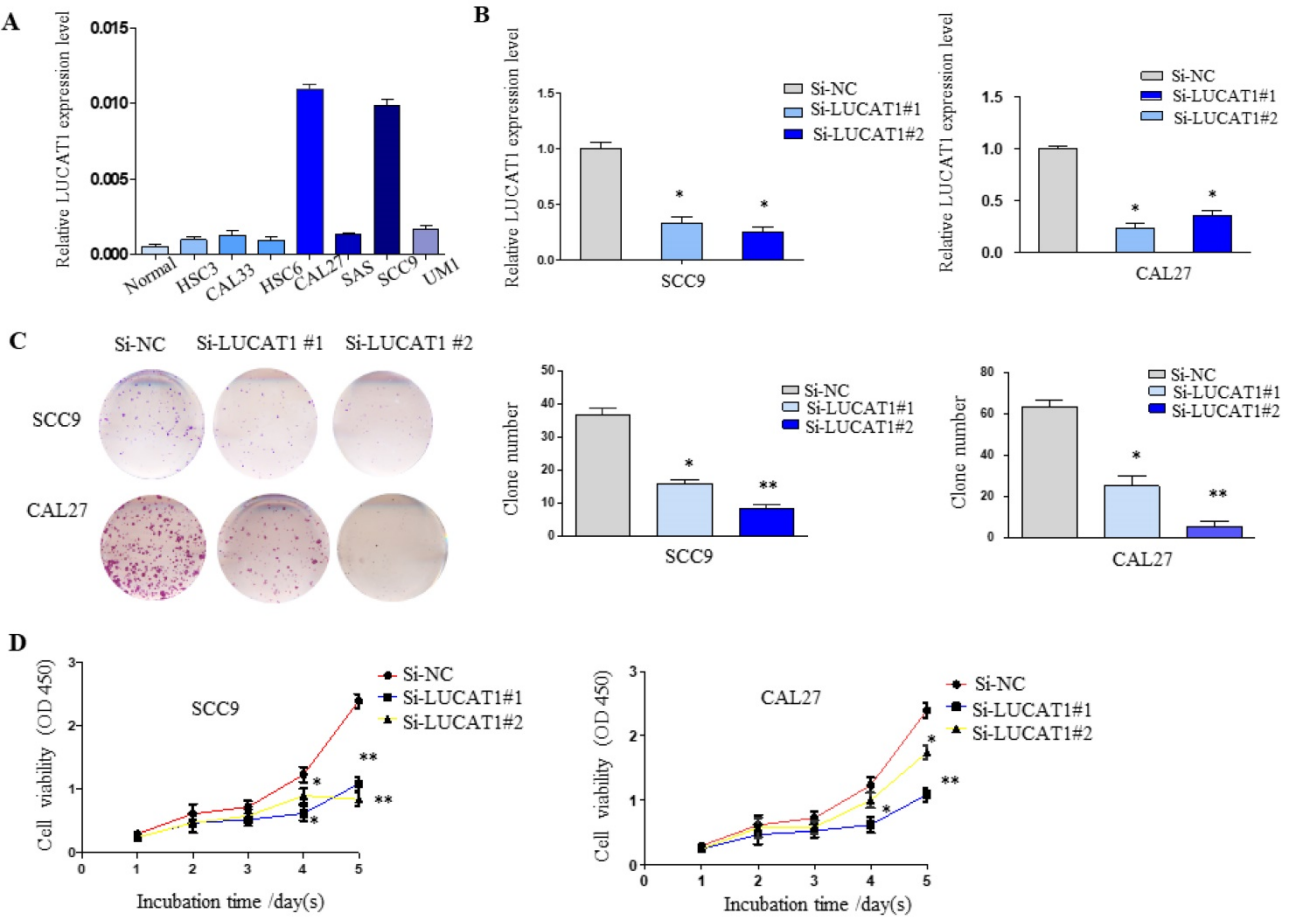
** LUCAT1 regulates TSCC cell growth *in vitro*. A.** Relative expression of LUCAT1 in TSCC cell lines. **B.** At 48 h after transfection, LUCAT1 expression was analysed by RT-qPCR. **C.** The results of colony formation in SCC9 and CAL27 cells transfected with siRNA against LUCAT1. **D.** At 48 h after transfection, a CCK-8 assay was performed to detect the proliferation of SCC9 and CAL27 cells. Error bars indicate the means ± S.E.M. *P < 0.05, **P < 0.01. ***P < 0.001.

To further understand the potential role of LUCAT1 in OSCC, RT-qPCR analysis was used to detect the expression of LUCAT1 in OSCC cell lines (normal oral mucosa epithelium cells, HSC3, HSC6, CAL27, CAL33, SAS, UM1 and SCC9). As shown in Figure 3A, LUCAT1 had the highest relative expression in CAL27 and SCC9 cell lines. Therefore, both CAL27 and SCC9 cells were chosen for further study. LUCAT1 was knocked down by transfection of LUCAT1 siRNA into CAL27 and SCC9 cells. After 48 h of transfection, RT-qPCR results showed that LUCAT1 expression was markedly reduced (Figure 3B).

To explore the effect of LUCAT1 on OSCC cell growth, cell proliferation was measured by colony formation and CCK-8 assays. The downregulation of LUCAT1 notably suppressed the colony formation abilities of SCC9 and CAL27 cells (Figure 3C). In addition, the growth curves generated by CCK-8 assays indicated that knockdown of LUCAT1 expression remarkably impaired SCC9 and CAL27 cell growth, which was consistent with the colony formation results (Figure 3D).

**Figure 4:**
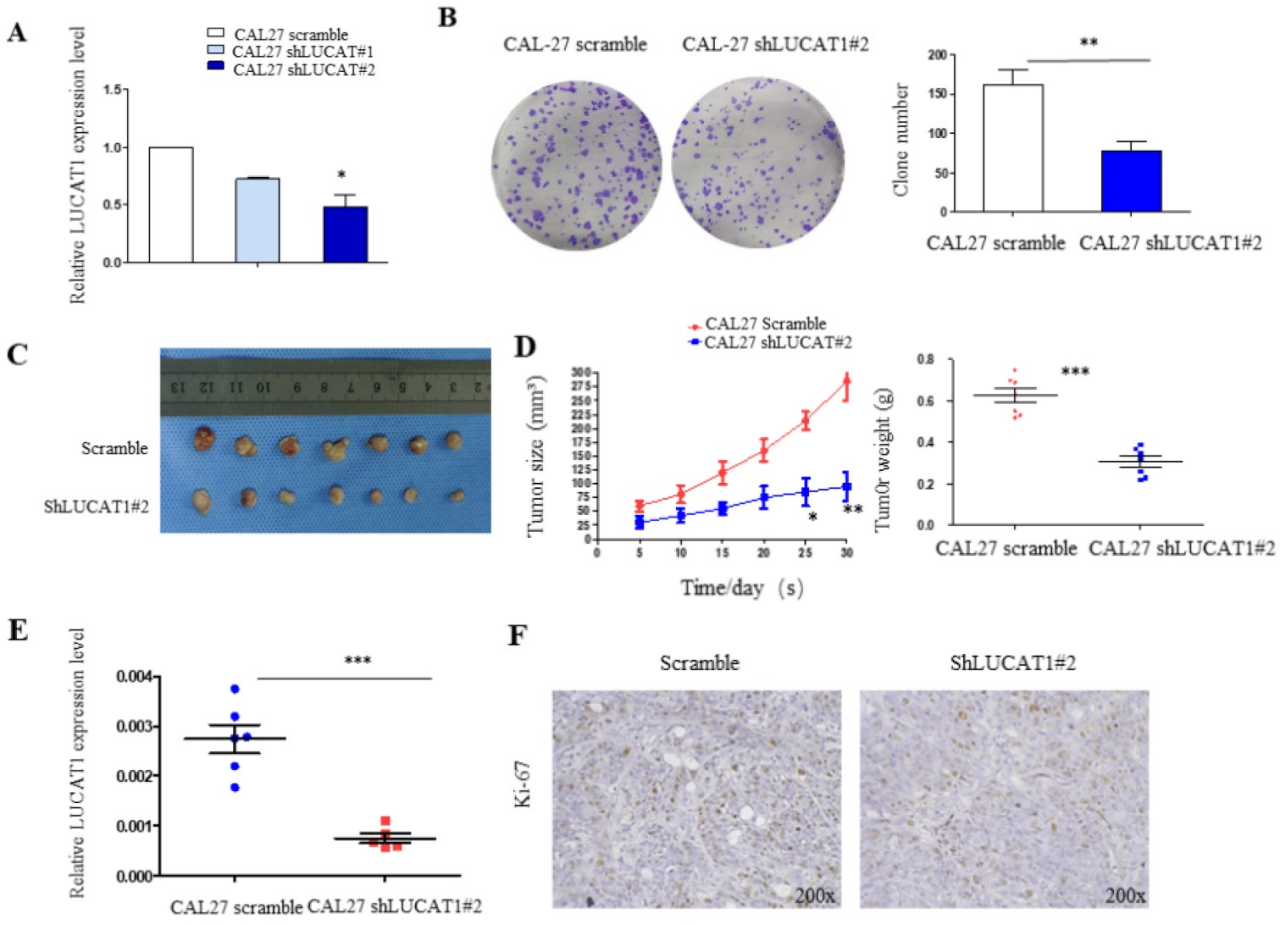
** The impact of LUCAT1 on tumourigenesis *in vivo*. A.** RT-PCR detection of the expression levels of LUCAT1 in CAL27 shLUCAT1 cells. **B.** Colony formation result in CAL27 shLUCAT1 cells. **C and D.** Scramble and shLUCAT1 were injected into nude mice (n = 7). The tumour volumes were calculated every 5 days after injection. The bars indicate SD. **E.** The tumour weights are shown as the means of tumour weights ± S.D. **F.** Histopathology of xenograft tumours. The tumour sections were subjected IHC staining using an antibody against Ki-67. Error bars indicate the means ± S.E.M. *P < 0.05, **P < 0.01. ***P < 0.001.

We used CAL27 shLUCAT1 cells for tumourigenesis *in vivo*. This analysis also revealed that shLUCAT1#2 had a higher silencing efficiency than shLUCAT1#1 (Figure 4A), so we chose shLUCAT1#2 for further experiments. As shown in Figure 4B, knockdown of LUCAT1 expression markedly inhibited cell proliferation compared to that in the control cells.

### Knockdown of LUCAT1 inhibited OSCC cell proliferation *in vivo*

To determine the effects of LUCAT1 on tumourigenesis *in vivo*, we subcutaneously injected CAL27 cells transfected with either shLUCAT1#2 or scrambleinto the flanks of 5-week-old nude mice. Five days after injection, all mice developed xenograft tumours at the injection site. We measured the xenograft diameters and weighed mice every 5 days and found that tumour growth in the shLUCAT1 group was measurably slower than that in the scramblegroup (Figure 4C). Moreover, the average tumour weight and volume were distinctly lower in the shLUCAT1 group than in the scramble group (Figure 4D). RT-qPCR results showed that LUCAT1 was significantly higher in tumours of the scramble group than the shLUCAT1 group (Figure 4E). Additionally, we found that the xenografts generated by CAL27 shLUCAT1 cells had lower Ki-67 expression than the xenografts generated by CAL27 scramblecells (Figure 4F). As shown in Table 4, the difference is statistically significant. These results provide further evidence that the downregulated expression of LUCAT1 was significantly correlated with the decreased proliferative capacity of OSCC cells *in vivo*.

### VitD inhibited the MAPK signalling pathway of OSCC by inhibiting the expression of LUCAT1

**Figure 5:**
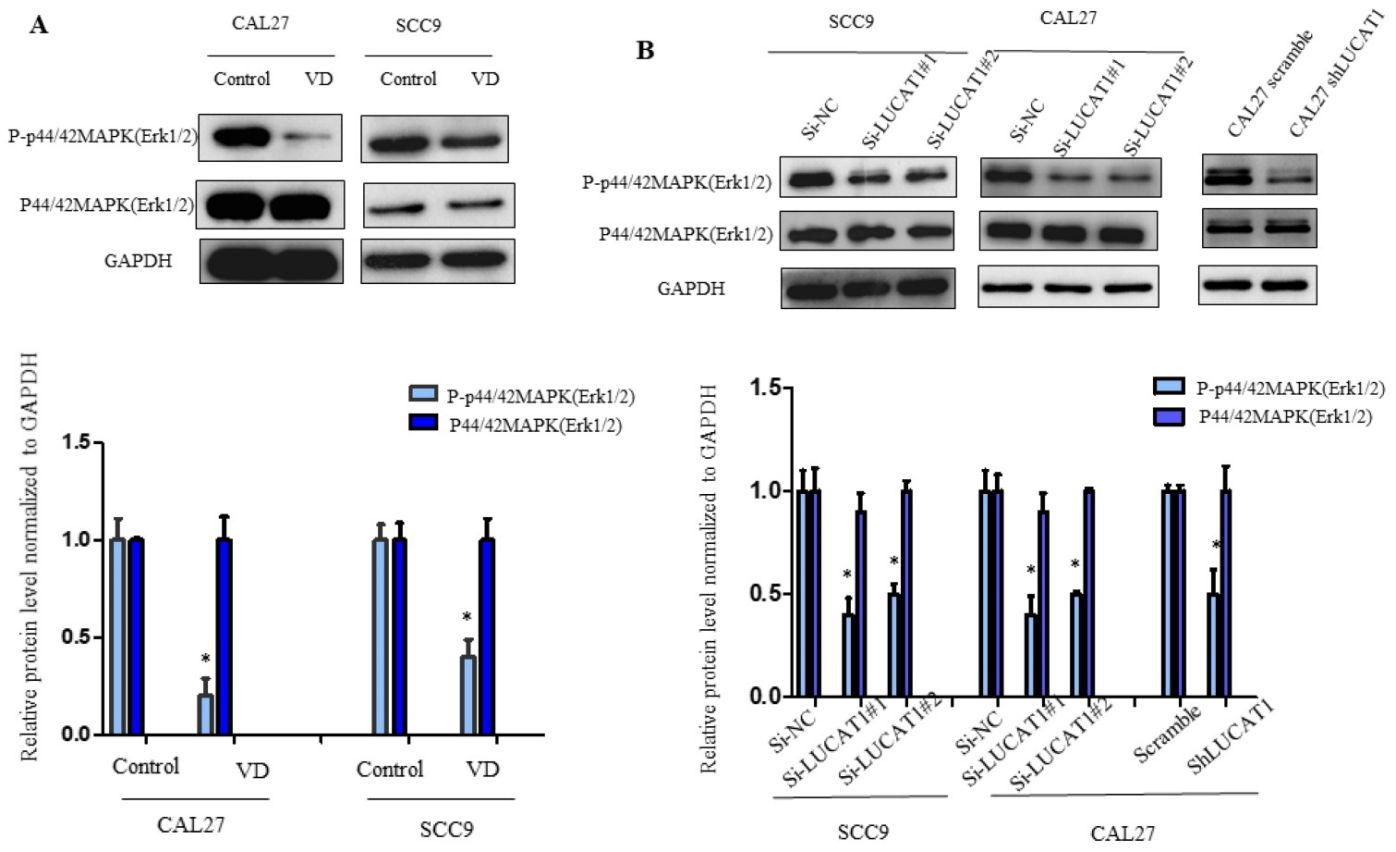
** Effect of vitamin D and LUCAT1 on the MAPK signalling pathway. A.** The protein level of the MAPK signalling pathway was examined after vitamin D treatment. **B.** Detection of MAPK signalling pathway protein levels after low expression of LUCAT1.

It has been reported that VitD inhibits MAPK signalling pathway activation 16. The MAPK signalling pathway participates in the physiological functions of various cancer cells *in vivo*, including proliferation, apoptosis and differentiation 17. To detect the effect of VitD on the MAPK signalling pathway in OSCC cells, we detected the key proteins ERK1/2, p38 and JNK. We found that phosphorylation of ERK1/2 in OSCC cells treated with VitD was significantly inhibited, while total ERK1/2 was not changed (Figure 5A). Therefore, we further confirmed that MAPK was an important regulator of VitD. Similarly, we tested whether LUCAT1 also affects the MAPK signalling pathway. By exploring possible regulatory mechanisms downstream of LUCAT1 on MAPK, we found that MAPK phosphorylation was significantly inhibited and affected the activation of the MAPK signalling pathway in the si-LUCAT1 group and shLUCAT group (Figure 5B).

**Figure 6:**
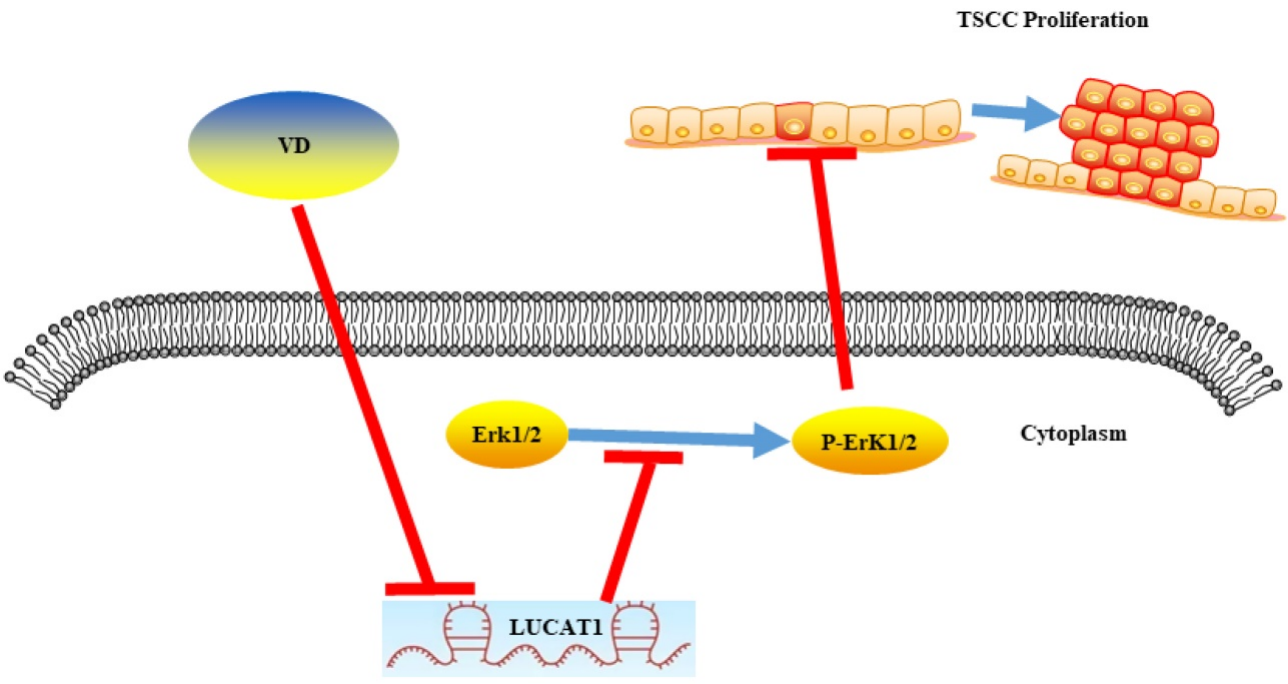
A schematic model showing that vitamin D inhibited the growth of OSCC cells through the LUCAT1-MAPK signalling pathway.

In conclusion, LUCAT1 was a key molecule responsible for proliferation of OSCC. Vitamin D inhibited MAPK signalling pathway activation by suppressing LUCAT1, which suppressed the proliferation of oral cancer (Figure 6).

## Discussion

Vitamin D has been confirmed to have antitumour effects in previous studies. Evidence from epidemiologic, preclinical, and clinical studies indicates that VitD might have usability as a therapeutic agent in cancer patients 18. VitD has an antitumour effect through modulation of inflammation, cell proliferation, cell differentiation, angiogenesis, invasive and metastatic potential, and apoptosis 19. Previously, it has been reported that VitD3 and 13-cis retinoic acid have equipotent antiproliferative effects on tongue squamous cell carcinoma (SCC-25) cells 20. Additionally, 1,25(OH)2D3 has been shown to inhibit the growth of HNSCC cells through the upregulation of cell cycle inhibitor p18 expression 21. We previously found that VitD promotes the cisplatin sensitivity of oral squamous cell carcinoma by inhibiting LCN2-modulated NF-κB pathway activation through RPS3 11. This study further demonstrated that VitD can significantly inhibit the proliferation of OSCC; therefore, VitD may be used as a drug to prevent and treat oral cancer in clinical practice.

Recently, efforts have been dedicated to elucidating the biological functions and clinical impact of long noncoding RNAs, and studies have revealed that lncRNAs might be novel biomarkers for several types of human cancers, including OSCC 22. It has been reported that some lncRNAs play an important role in the development of OSCC 23, 24. In this study, we determined that VitD inhibited OSCC cell proliferation through the regulation of LUCAT1. We examined the expression levels of LUCAT1 in OSCC clinical specimens and cell lines and found that LUCAT1 was significantly increased in OSCC samples. High LUCAT1 expression was an independent prognostic factor for liver cancer 25. Therefore, LUCAT1 can be used as a biological indicator and a prognostic factor of OSCC, and LUCAT1 is a clinical marker that can be used for patient management plans to improve treatment.

Esma Karkeni et al. demonstrated for the first time that VitD modulates the expression of miRNAs in adipocytes *in vitro* and in adipose tissue *in vivo* through its impact on the NF-κB signalling pathway 26. We investigated the impact of VitD on lncRNA expression in OSCC using high-throughput sequencing, and we identified for the first time lncRNA LUCAT1 as a potential VitD target. How LUCAT1 is activated directly by VitD should be further explored.

Lung cancer-associated transcript 1 (LUCAT1) was first reported to be involved in smoking-related lung cancer. It has been reported that LUCAT1 has an antitumour effect by modulating miRNA expression and signalling pathways in clear cell renal cell carcinoma, bladder cancer, colorectal cancer, hepatocellular carcinoma, breast cancer and cervical cancer 27.

To identify molecular mechanisms regulating this process, several approaches were combined. Previous studies have shown that LUCAT1 promoted proliferation and invasion in clear cell renal cell carcinoma cells through the AKT/GSK-3β signalling pathway 28. To the best of our knowledge, the mitogen-activated protein kinase/extracellular signal-regulated kinase (MAPK/ERK) pathway has been reported to be associated with cell proliferation, differentiation, migration, senescence and apoptosis. Therefore, a western blot experiment was performed showing that low expression of LUCAT1 can significantly inhibit the activation of the mitogen-activated protein kinase/extracellular signal-regulated kinase (MAPK/ERK) pathway, thus affecting the proliferation of OSCC. Based on our results, it is reasonable to conclude that LUCAT1 inhibits the MAPK signalling pathway in tongue squamous cell carcinoma.

Despite advances in surgical techniques and postoperative radiotherapy, the overall 5‐year survival of OSCC patients has not significantly improved in the last 50 years. Therefore, new therapies for OSCC are urgently needed. To find a more effective treatment, in addition to early surgery-radiotherapy and chemotherapy sequence treatment, EGFR inhibitors and COX-2 inhibitors photodynamic therapy, as well as FOXM1 and PD-L1 inhibitors, have been proposed 29. However, all have led to nonspecific cell death. VitD can reduce the expression of the tumour-promoting factor LUCAT1 and inhibit the proliferation of OSCC. VitD supplementation should be a better choice than using LUCAT1 inhibitors to inhibit the proliferation and progression of OSCC. Further clinical trials are certainly needed for the dosage and time-course effects.

## Conclusion

In summary, VitD treatment significantly inhibited the expression of LUCAT1, downregulated MAPK activation, and inhibited OSCC growth. These results reveal the therapeutic potential of VitD for OSCC treatment, prevention and delay of the onset of recurrence. Thus, improvement of VitD status with sensible sun exposure, VitD supplementation and ingesting foods containing VitD is a reasonable strategy to reduce the risk of malignancy. In addition, LUCAT1 is expected to be a new target for biotherapy.

## Supplementary Material

VitD-regulated lncRNAs.

## Figures and Tables

**Table 1:** The siRNA sequences

Gene	Sense (5'-3')	Antisense (5'-3')
LUCAT1#1	CCCAUCAGAAGAUGUCAGAAGAUAA	UUAUCUUCUGACAUCUUCUGAUGGG
LUCAT1#2	CAAGCUCUUGCAGUCAACAAGAACU	AGUUCUUGUUGACUGCAAGAGCUUG

**Table 2:** The primer sequences

Gene	Forward	Reverse
LUCAT1	GCTCGGATTGCCTTAGACAG	TGCCAAGGTCCCATAAGAGT
GAPDH	GAGTCAACGGATTTGGTCGT	GACAAGCTTCCCGTTCTCAG

**Table 3:** Relationship between lncRNA LUCAT1 expression and clinicopathologic parameters in 46 TSCC patients

Characteristics	Number (%)	LUCAT1 expression	*P* value
**Tumour size**			
T1	6 (13.04%)	0.0017 ± 0.0019	
T2	16 (34.78%)	0.0064 ± 0.0030	
T3	12 (26.09%)	0.1510 ± 0.0515	
T4	12 (26.09%)	0.8810 ± 0.1055	0.0013
**Differentiation**			
Well	10 (21.74%)	0.0122 ± 0.02125	
Moderate	23 (50%)	0.0432 ± 0.08681	
Poor	13 (28.26%)	0.0180 ± 0.00994	0.1413
**Lymphatic metastasis**			
Yes	21 (45.65%)	0.0821 ± 0.1034	
No	25 (54.55%)	0.0287 ± 0.0629	0.0617

**Table 4:** Statistical analysis of Ki-67 expression in xenografts

Group	Ki67 expression		*P* value
	High	Low	
Scarmel	6	1	
Sh-LUCAT	1	6	0.029

## Abbreviations

VitD: Vitamin D
OSCC: Oral squamous cell carcinoma
TSCC: Tongue squamous cell carcinoma
lncRNAs: Long noncoding RNAs
MAPK: Activation of the mitogen protein kinase
LUCAT1: Lung cancer associated transcript
RT-qPCR: Quantitative real time polymerase chain reaction
SiRNA: Small interfering RNA
